# Supplementation of Live Yeast, Mannan Oligosaccharide, and Organic Selenium during the Adaptation Phase of Newly Arrived Beef Cattle: Effects on Health Status, Immune Functionality, and Growth Performance

**DOI:** 10.3390/antibiotics10091114

**Published:** 2021-09-15

**Authors:** Silvia Grossi, Matteo Dell’Anno, Luciana Rossi, Riccardo Compiani, Carlo Angelo Sgoifo Rossi

**Affiliations:** Department of Health, Animal Science and Food Safety “Carlo Cantoni” (VESPA), Università degli Studi di Milano, 26900 Milan, Italy; matteo.dellanno@unimi.it (M.D.); luciana.rossi@unimi.it (L.R.); riccardo.compiani@gmail.com (R.C.); carlo.sgoifo@unimi.it (C.A.S.R.)

**Keywords:** antimicrobial resistance, nutraceuticals, beef cattle, immunity

## Abstract

The effect of a nutraceutical mixture, based on live yeast (LY), mannan oligosaccharides (MOS) and organic selenium (Se) on health status, as well as immune functionality and growth performance in the fattening of newly received beef cattle, was evaluated. A total of 1036 Charolaise heifers were allocated into two experimental groups: (i) control group, without any nutraceutical support (*n* = 487; initial weight = 325 ± 21 kg); and (ii) treatment group, supplementation during the first 30 days, with LY (5 g/head/day), organic Se (3 mg/head/day), and MOS (10 g/head/day) (*n* = 549; initial weight = 323 ± 23 kg). The incidence of bovine respiratory disease (BRD) and other health issues was monitored, as well as the mortality rate. Blood samples were taken at d_0_ and d_30_ to evaluate the immune functionality and the inflammatory status. Growth performances, feces chemical composition, and carcass characteristics were recorded. The BRD occurrence tended to be reduced (*p* = 0.06) in the Treatment group. The BHV-1 antibody production after vaccination was significantly improved (*p* = 0.031), as well as the bactericidal activity (*p* = 0.0012) in the Treatment group. No differences were found in the inflammatory status parameters. The final weight (*p* = 0.006) and the average daily gain at d_30_ (*p* < 0.0001) were significantly improved by the treatment. No differences were found in terms of carcass characteristics, while the fecal content of NDF (*p* < 0.0001), ADF (*p* = 0.0003), and starch (*p* < 0.0001) were significantly reduced by the treatment. The result of the present study suggests that the nutraceutical mixture used can support the animal’s immune systems, improving its ability to react against pathogens, as well as feed efficiency and growth performances during the whole fattening period.

## 1. Introduction

Globally, 700,000 people die each year due to infection with antibiotic resistant organisms (AROs), prospecting to rise up to 10 million deaths per year in 2050. At the European level, antimicrobial resistance (AMR) is estimated to be responsible for 33,000 deaths per year. Moreover, infection with antimicrobial resistant pathogens can lead to serious illnesses and increased hospitalization rate, with a stronger pressure on healthcare systems’ stability, efficiency, and economy [1]. For instance, just in Europe, it has been estimated that AMR costed more than 9 billion euros per year [1,2].

AMR occurs when microorganisms, such as bacteria, viruses, fungi, and parasites, become resistant to the medications normally used to fight them [3]. The acquisition of resistance characters can be the result of natural selective mechanisms as well as a process induced by human-related factors, such as misuse and abuse of antimicrobials mainly in human but also in veterinary medicine [4].

Considering the importance of the problem, the European community is moving towards the implementation of new monitoring systems and regulations regarding the use of antibiotics in both human medicine and animal husbandry [2,3,4,5].

Indeed, even if the antimicrobial consumption in food-producing animals has decreased and is now lower than in humans, as reported by the latest European Centre for Disease Prevention and Control (ECDC) and European Food Safety Authority (EFSA) reports, the use of antibiotics in the farming sector is coming under increasing scrutiny, especially when used for prevention of disease, in the form of prophylaxis or metaphylaxis, in particular with antimicrobials commonly used in human medicine [1,5,6]. Their use should be limited, and allowed only if strictly necessary, as will be underlined also in the new European regulations [7]. Specifically, the attention should be focused on the molecules defined as “highest priority critically important antimicrobials” (HPCIAs) by the World Health Organization (WHO) [7].

In livestock farming, swine and poultry accounted for the highest use of antibiotics, while cattle are in third position in terms of milligrams per population correction units (PCU) (172 mg/PCU, 148 mg/PCU, and 45 mg/PCU, respectively) at a global level [8].

However, within intensive beef cattle farming, there are still stages of the rearing period that require higher quantities of antimicrobials.

In the beef cattle system, based on the fattening of weaned cattle imported from pasture areas, the arrival stage represents the most critical period for animal health, welfare and also antibiotics use [9]. Newly received cattle are exposed to several stressors, such as weaning, long-distance transport, mixing, feed and water restrictions, and adaptation to new environmental and feed conditions [10]. All these factors contribute to pathogen colonization and proliferation as well as to a stress-related reduced immune functionality, leading to the spread of diseases, especially bovine respiratory disease (BRD).

Besides being one the most common cause of morbidity and mortality, BRD is also associated with an overall reduction in productivity during the whole rearing cycle [9,10,11]. Animals that suffer from BRD in the first days after the arrival show poor growth performance that can result in a reduction in the average daily gain over the entire fattening period. For this reason, higher quantities of antibiotics are often used in the arrival stage, both to treat sick subjects but also to prevent the spread of the problem [9].

Actually, a reduction in the use of antimicrobials is required, especially in terms of prophylaxis and mass treatments [5]. Alternative strategies must be developed to improve the immune functionality, lowering the negative effects of stress and improving the animal’s intrinsic ability to cope with diseases. In this direction, nutrition can have a proactive role. Beyond satisfying and respecting all the nutritional needs, nutrition can become a vehicle of functional feed ingredients, which sustain the health status and reduce the risk of pathologies [9,10,11,12].

In fact, in the last few years, natural products and organic minerals are increasingly being studied in animal nutrition, both in monogastric, such as pigs [12] and broilers [13], as well as in ruminants, with specific attention on the most critical stages, such as the adaptation period in beef cattle [9,10,11,12,13,14] and transition days in dairy cows [15].

Most studies carried out in beef cattle focused their attention on live yeast (LY) [16], yeast-derived products such as mannan oligosaccharides (MOS) [14], and organic selenium (Se) [9].

Live yeasts are probiotics, mainly belonging to the *S. cerevisiae* species, currently used in livestock production for a variety of reasons encompassing performance enhancement and overall benefits to animal health and welfare [17].

Mannan oligosaccharides (MOS) are prebiotics composed of complex carbohydrate molecules, derived from the outer cell wall of *S. cerevisiae*, known as elements capable of improving animal productivity through a better gastrointestinal and ruminal health [18].

Selenium (Se) is a mineral involved in many different metabolic functions, principally related to the maintenance of the body redox balance and antioxidant defences, thus also regulating the immune system [9,10,11,12,13,14,15,16,17,18,19]. The ability of Se to improve the immune response in farm animals is well documented [20]. In animal feed, selenium can be integrated both in inorganic or organic forms. The organic sources, in which selenium is contained mainly in the form of selenomethionine, are characterized by a higher bioavailability level [19,20,21].

Indeed, the hypothesis of the present study was that dietary supplementation of live yeast, mannan oligosaccharides, and organic selenium can positively affect the health status, the immune functionality, and the performance of fattening beef cattle in the arrival period, taking also into account the actual lack of specific studies in bibliography.

The aim of the present study was to evaluate the potential effect of a nutraceutical mixture, composed by live yeast (LY) (ActiSaf–Phileo Lesaffre Animal Care, Marcq-en-Barœul, France), mannan oligosaccharides (MOS) (SafMannan–Phileo Lesaffre Animal Care, Marcq-en-Barœul, France), and organic selenium (Se) (SelSaf–Phileo Lesaffre Animal Care, Marcq-en-Barœul, France) on health status, immune functionality, and growth performance in newly arrived beef cattle

## 2. Results

### 2.1. Health Status

Data on health status and incidence of pathologies in the two groups are reported in Table 1. The inclusion of the nutraceutical mixture significantly reduced the morbidity for bovine respiratory disease (BRD) (51.2% vs. 60.2%, respectively, in the Treatment and Control groups) (*p* = 0.003). Moreover, a significantly lower percentage of animals relapsed after the first pull of the pathology in the Treatment compared to the Control group (9.6 vs. 16.3) (*p* = 0.001).

Neither mortality (0.7 vs. 0.8) (*p* = 0.864), incidence of lameness (0.0 vs. 0.2) (*p* = 0.288), and number of animals moved to the infirmary pen (0.0 vs. 0.2) (*p* = 0.864) were influenced by the treatment.

### 2.2. Hematological Parameters Related to Immune Function

Data on blood parameters, indicators of immune functionality, and inflammatory status are reported in Table 2. The inclusion of the nutraceutical mixture significantly improved the specific immune reactivity, increasing the production of antibodies specific for bovine herpes virus 1 (BHV-1) after the vaccination in treated animals compared to the control ones (0.71 vs. 0.59) (*p* = 0.031). Furthermore, the non-specific immune reactivity was better in the Treatment group, with a statistically higher serum bactericidal activity compared to the Control at d_30_ (84.30 vs. 80.86) (*p* = 0.0012). Conversely, at d_30_, the levels of γ-interferon were not influenced by the treatment (*p* = 0.302).

Regarding the indicators of the inflammatory status (haptoglobin, lipopolysaccharide binding protein, and interleukin 6), no statistically significant differences were found between groups.

### 2.3. Growth and Slaughtering Performance

Data on growth performance are reported in Table 3. The inclusion of the nutraceutical mixture, based on LY, MOS, and organic Se, in the first 30 days after the arrival significantly improved growth performance. Indeed, treated animals showed a significantly higher weight at the end of the fattening period (519.01 ± 32.40 vs. 514.81 ± 24.81 in the Control) (*p* = 0.006), as a result of a higher growth in the first month in the Treatment compared to the Control group (0.896 ± 0.18 vs. 0.803 ± 0.14) (*p* < 0.0001). Considering the hole period of 186 days, the trend seemed to be tending to statistical significance. Specifically, the difference between the two groups was 93 g/head/day during the adaptation stage and 31 g/head/day during the whole fattening period.

Data on slaughtering performances and carcass characteristics are reported in Table 4. Neither the slaughtering performance (cold carcass weight and dressing percentage) nor carcass characteristics were affected by the treatment (*p* > 0.05).

### 2.4. Chemical Composition of Feces

Data on the fecal chemical composition are reported in Table 5. The inclusion of the nutraceutical mixture based on LY, MOS, and organic Se in the first 30 days after the arrival significantly enhanced feed efficiency and digestibility. In fact, the content of NDF (*p* < 0.001), ADF (*p* = 0.0003), and starch (*p* < 0.001) in the feces of treated animals was significantly reduced at d_30_.

## 3. Discussion

The ever-increasing demand for a reduction in the consumption of antimicrobials has driven producers to seek more innovative and “natural” approaches in beef cattle farming. These innovative strategies can include specific feed supplements, such as live yeast (LY), yeast-derived products such as mannan oligosaccharides (MOS), as well as organic minerals such as organic selenium (Se), with nutraceuticals properties able to provide health benefits during the most critical rearing phases.

The onset of pathologies, especially bovine respiratory disease (BRD), during the critical adaptation period is also correlated with poor growth performance during the entire fattening period, with an overall reduction in average daily gain of nearly 150–200 g/day, with peaks of over 300 g in case of multiple relapses [10]. The primary reason of this reduction in growth performance is related to a decreased feed intake, due to an altered health status. Furthermore, in this situation, the energy demand can increase by about 10–30% to support the immune system activity, with some estimates even higher at 55% compared to the metabolizable energy needed usually, mainly in the form of glucose [22,23,24]. Indeed, the activation of the immune system required at least 0.5 kg of additional glucose in ruminants, with peaks of about 1 kg within a 12 h period [25,26]. Consequently, part of the feed-derived energy is shifted toward the activation of the immune system instead of being used for growth [23,25,26].

Nutraceutical nutrients, such as LY, MOS, and organic Se, may act as important support for newly arrived beef cattle, due to both their immunological and functional properties. Indeed, they can act both directly on the specific and non-specific immunity as well as indirectly modifying the metabolism, leading to a better release of glucose and energy precursors from the feeds, usable by the immune system [23].

The results of the present study underlined a positive effect of the treatment on health status, with reduced mortality and morbidity rate, and with a significant reduction in the incidence of BRD. The present findings are in line with the previous researches of Keyser et al. (2007) and of Fink et al. (2014) which reported a decrease in morbidity rate, in recovery time from BRD infection, and in the number of antibiotic treatments per animal, in beef cattle fed with LY during the arrival phase [16,17,18,19,20,21,22,23,24,25,26,27]. Moreover, Sgoifo et al. (2017) found a reduced incidence of BRD in newly arrived beef cattle fed with organic Se [9].

This improvement in the health status can be explained by a better immune function. In fact, the result of the present study highlighted an increased antibodies production and serum bactericidal activity in treated animals, in line with previous findings of different researches [9,14,15,23]. The increase in antibody production can be related to a higher lymphocyte B activity, supported by a higher blood level of Se, in the form of selenomethionine, present both in the organic Se as well as in LY, and of β-glucans [9,19,23]. Consequently, the activity of glutathione peroxidase (GSH-Px) was increased, which is needed to maintain a correct functionality of the lymphocyte [9,28]. Indeed, accumulation of free radicals within the lymphocytes, especially during an inflammatory response, cause these to be less effective. This is one of the reasons because a better antioxidant status can lead to a greater production of antibodies [28]. An increased level of antibodies was detected also in the blood of dairy cows fed with LY or yeast-derived products, with a high content of β-glucans during the transition period, testifying their beneficial role in stressful situations [29,30].

Furthermore, components of LY and yeast-derived products, especially β-glucans, have been shown to enhance non-specific immune function through improving the actions of macrophages, neutrophils, and eosinophils and increasing the clearance of pathogens, exerting a better protection activity against invading pathogens [31]. Specifically, yeast and yeast-derived products may be involved in the synthesis and release stimulation of the pro-inflammatory cytokines from macrophages and others such as interleukins (IL-1, IL-2, and IL-6) [17,18,19,20,21,22,23]. The results of Burdick-Sanchez et al. (2013) highlighted a significantly lower production of IL-6 in beef heifers feed with yeast-derived product, during the arrival period after an LPS endotoxin challenge, compared to control ones [32]. Furthermore, the increase in rectal temperature after the challenge was lower in treated heifers as a result of a better non-specific immune functionality [32]. The efficacy of β-glucans on non-specific immunity was highlighted also in transition dairy cows, where the direct administration of them markedly reduced serum levels of pro-inflammatory cytokines, as a result of an improved immune functionality and a reduced inflammatory status [30].

Even if the result of the present study did not find significant changes in terms of both γ-interferon levels and inflammatory status (haptoglobin, lipopolysaccharide binding protein, and interleukin 6 levels), the improved serum bactericidal activity, detected in the treatment group, highlighted a better functionality of the non-specific immune defences. In normal conditions, serum bactericidal activity has to be higher than 90% [9]. Due to the stressors associated with transport, animals showed a lower serum bactericidal activity than the optimal threshold on arrival, and during the adaptation period. In the present conditions, the serum bactericidal activity values were significantly higher in treated animals because of a lower effect of stressors on health status and better non-specific immunity at d_30_.

As previously stated, nutraceuticals, such as LY and yeast-derived products, may also exert an indirect effect on the immune system through an alteration of the metabolism, improving the energy availability in the form of glucose [23]. In fact, the blood glucose levels were significantly higher after a LPS endotoxin challenge in beef steers fed with different yeast-derived products [33,34]. These two studies suggest that supplementation of yeast-derived products may improve energy availability during an immune challenge, which may be beneficial to allow a more rapid resolution. Additionally, lactating dairy cows supplemented with LY or yeast culture had greater serum glucose compared to control ones [35].

There are several possible explanations for the increase in glucose concentrations, mainly related to the stabilizing and promoting effects that LY and yeast-derived products can exert at the ruminal level. In fact, LY and its derivates act at the ruminal level as an oxygen consumer, decreasing the redox potential of the rumen, thus promoting a more favorable environment for the development of the ruminal microflora, mainly cellulose consumers, and lactic acid-utilizing bacteria [36]. Increases in fibrolytic, cellulolytic, and lactic acid-utilizing bacteria helped to increase and stabilize the ruminal pH, thus reducing the risk of acidosis and also improving the functionality of the other bacteria, such as the propionate producers [36]. Consequently, the availability of substrates, such as acetate, increased the glucose levels as a result of the maximized digestion of fiber-based feedstuffs [23,24,25,26,27,28,29,30,31,32,33,34,35,36,37]. Moreover, the higher level of propionate, consequent to a better functionality of the other bacteria strains in the rumen, may also play a role in increasing serum glucose concentrations in cattle [23,24,25,26,27,28,29,30,31,32,33,34,35,36,37,38]. In fact, higher propionic acid concentrations can lead to an increased glucogenic potential of the diet of dairy cows fed with yeast derived products [38]. Furthermore, Crossland et al. (2018) found an increase availability of metabolizable and digestible energy in finishing steers fed with active dry yeast, as a result of a higher feed efficiency at the ruminal level [39].

The results of the present study agreed with those findings. Indeed, the reduced presence of NDF, ADF, and starch in the faeces of treated animals can be explained by better and higher digestibility at the ruminal level, considering that the nutritional management was the same in the two groups. Moreover, the higher availability of volatile fatty acids (VFA), highlighted in previous bibliographical studies, can also explain the better growth performances highlighted in the present study in the treatment group, as a result of a feed digestibility and utilization [36,37,38,39]. In fact, the results indicate an overall positive effect of the inclusion of the nutraceutical mixture also on growth performance, both specifically in the adaptation as well as considering the entire fattening period. Those data are in line with the findings of previous researches carried out in beef cattle which have evaluated the different compounds separately [9,14,40]. Indeed, Smith et al. (2020) found a 4.9% increase in ADG in beef steers fed with LY during a 47-day adaptation period [40]. Tassinari et al. (2013) found a 3.6% increase in ADG in Blonde Aquitaine bulls fed with MOS during the first 48 days of fattening [14]. Moreover, Sgoifo Rossi et al. (2017) have shown an improvement in growth performance in beef cattle supplemented with organic Se, compared to inorganic, after a long-lasting Se supplementation and this might be explained by the higher bioavailability of organic Se having a positive effect on the health and antioxidant status [9].

## 4. Materials and Methods

### 4.1. Animal, Groups and Animal Care

The study was performed in a commercial farm, representative of the typical Italian intensive beef cattle farming system.

A total of 1036 newly received Charolaise heifers, imported from France, were enrolled. At three days after arrival (d_0_), all the animals were individually weighed and evaluated for the conformation, assessed on a 5-point scale (1: profiles straight and poor muscle development; 2: profiles between whole straight to low convex and medium muscle development; 3: profiles low convex and good muscle development; 4: profiles on the whole convex and very good muscles development; 5: all profiles convex and exceptional muscles development) [41]. The animals were grouped by conformation and body weight, and randomly assigned to two balanced experimental groups, differing for the inclusion in the diet of the nutraceutical support: (i) traditional nutritional management (Control group) (*n*. 487; initial weight 325 ± 21 kg); and (ii) traditional nutritional management integrated, during the first 30 days after arrival, with a nutraceutical mixture composed by live yeast (LY) (5 g/head/day) (ActiSaf), organic selenium (Se) (3 mg/head/day) (SelSaf), and mannan oligosaccharides (MOS) (10 g/head/day) (SafMannan) (Treatment group) (*n*. 549; initial weight 323 ± 23 kg). The product was administered for the first 30 days after the arrival, while the animals were followed for the entire fattening period (187 days).

The heifers were housed in a close barn with 104 pens with 9–10 animals each (3.5 m^2^ each), with fully slatted floor.

### 4.2. Feeding Protocol

The two groups received the same feeding plan (Table 6). The feed was administered *ad libitum* in form of total mixed ration (TMR), and delivered once a day in the morning by a feed mixer wagon, provided with electronic scale to weigh the inclusion of each ingredient and the TMR unloaded. The TMR was studied to meet the growth needs of the animals, as required by the Nutrient Requirement Council [42]. The fattening period was divided into 2 subperiods to better fit the different needs of every growing stage: arrival (0–30 days) and fattening (30–186 days).

The two experimental groups differed for the administration of the nutraceutical mixture in the Treatment group during the first 30 days. The pool, included directly in the mineral mix, was formulated to supply: (i) 0.0278 g/g of mineral mix on dry matter (d.m.) basis of LY, resulting in a total of 5 g/head/day; (ii) 0.0166 mg/g of mineral mix on dry matter (d.m.) basis of organic Se, resulting in a total of 3 mg/head/day; and (iii) 0.056 g/g of mineral mix on dry matter (d.m.) basis of MOS, resulting in a total of 10 g/head/day.

In the control group a placebo, (15.00 g/head/day) of wheat bran was added to the mineral mix to maintain the same dry matter administered. The mineral mix differed between the two groups only for the first 30 days of adaptation.

### 4.3. Experimental Parameters

#### 4.3.1. Health Status

From day 0 to day 186, general health evaluations were conducted twice a day, with a direct examination of all the animals by the farm veterinary and qualified animal health care staff. Any case of morbidity and mortality were recorded, as well as the number of animals that needed to be moved to the infirmary pen, together with the motivation, with specific attention on the incidence of bovine respiratory disease (BRD) and lameness. Sick animals were considered affected by BRD if the rectal temperature was ≥40.0 °C and if both depression and respiratory scores differed from the normal health status (score 0 of Baggott et al., 2011) [43]. BRD new events were recorded as first pull. Furthermore, all the relapses were recorded as I°, II°, or III° relapse, according to how many times one animal showed signs of BRD after the first episode. Sick animals received concurrent medications according to the facility procedures and returned to their study pens or moved to infirmary when necessary.

#### 4.3.2. Blood Samples and Hematological Parameters to Evaluate Immune Function

At d_0_ and d_30_, blood samples were taken from 30 animals per group to evaluate some serum indicators of immune functionality and inflammatory status. Blood samples were collected by jugular venipuncture into 10 mL EDTA tubes and 10 mL no additive tubes (Venoject^®^, Terumo Europe N.V., Leuven, Belgium) and immediately placed on ice. Then, the serum was extracted by centrifugation at 3000 g for 10 min at 4 °C and stored at −20 °C.

The titration of BHV-1 vaccination antibodies with a BHV-1 serum neutralization test was evaluated as an indicator of specific immunity modulation. The BHV-1 serum neutralization test was performed according to OIE (2012) [44].

Non-specific immunity was evaluated with the analysis of the serum bactericidal activity and γ-interferon [45,46].

The inflammatory status was evaluated through the analysis of different acute stage proteins as haptoglobin (HPT), lipopolysaccharide binding protein (LBP), and interleukin 6 (IL6) [47,48,49].

#### 4.3.3. Growth and Slaughtering Performance

Individual body weight was recorded before morning feeding at three time periods, that covered the adaptation stage as well as the entire fattening period: on enrolment day (d_0_), at day 30 (d_30_), and at the day before slaughter (d_186_). The individual average daily gain (ADG) was then calculated for each period, namely from d_0_ to d_30_, from d_30_ to d_186_, and for the entire period from d_0_ to d_186_, using the following formula

(1) $$\mathrm{ADG}=\;\frac{{\mathrm{Weight}}_{f}-{\mathrm{Weight}}_{i}}{\mathrm{days}\;i-f}$$

where:

ADG = average daily gain (kg/head/day)

Weight f = final weight of each period

Weight i = initial weight of each period

Days i–f = days between the start and the end of each period

At the slaughterhouse cold carcass weights (CCW), carcass yield (CY), and SEUROP carcass rating scores (SCRS) were recorded for all the animals.

#### 4.3.4. Chemical Composition of Feces

At d_30_, before the morning feeding, fecal samples were collected on 30 animals per group. Then, all the different samples were pooled together to create only one sample per group. After the sampling procedures, the samples were sent to the laboratory, in order to determine nutrient excretion in the feces. Fecal samples were analyzed for analytical DM [50]. After this, each sample was chemically analyzed for crude protein (CP), using the Kjeldahl N methods, and for neutral detergent fiber (NDF) and acid detergent fiber (ADF), using combustion methods [50]. All the samples were also analyzed for ether extract [50]. Starch concentration was determined using an enzymatic hydrolysis of α-linked glucose polymers, as described by Rode et al. (1999) with modifications according to Vyas et al. (2014) [51,52].

### 4.4. Statistical Analysis

Statistical analyses were performed using SAS 9.3 (2010; SAS Institute Inc., Cary, NC, USA). The incidence of mortality, BRD, and its relapses and lameness were evaluated using a chi-square test.

The blood parameters were evaluated through an analysis of variance for repeated measure (ANOVA), with the fixed effect of treatment, time, and their interaction. The animal was considered as the experimental unit.

Each individual animal was also considered as the experimental unit for growth performance parameters, carcass characteristics, and slaughtering performance. A two-way analysis of variance (ANOVA) was performed considering the fixed effect of treatment, time, and their interactions. The same statistical analysis was performed for the evaluation of fecal chemical composition.

The significance level was set as *p* ≤ 0.05, while a *p* ≤ 0.001 represent a tendency toward significancy. Data are presented as least squared means and pooled standard error of the means (SEM).

## 5. Conclusions

The administration of a pool of nutraceuticals based on live yeast, mannan oligosaccharides, and organic selenium to beef cattle during the adaptation period resulted in a significant reduction in the bovine respiratory disease incidence, due to a significantly improved specific and non-specific immune defenses. Those data suggest an enhancement in the overall animal health and also a reduction in antibiotics use, in accordance with European regulations and with efforts to reduce the problem of antibiotic resistance. Moreover, growth performance and feed efficiency have been also optimized, as a result of the better general health status and more stable and healthier ruminal environment.

## Figures and Tables

**Table 1:** Health status in the two experimental groups.

	BRD ^1^					Other Pathologies		
Group	First Episode, % (*n*)	I° Relapse ^2^, % (*n*)	II° Relapse, % (*n*)	III° Relapse, % (*n*)	Total, % (*n*)	Infirmary ^3^, % (*n*)	Lameness, % (*n*)	Mortality, % (*n*)
Control ^4^	60.2 (293)	14.2 (69)	2.1 (10)	0.0 (0)	76.5 (372)	0.4 (2)	0.2 (1)	0.8 (4)
Treatment ^4^	51.2 (281)	7.8 (43)	1.3 (7)	0.5 (3)	60.8 (334)	0.0 (0)	0.0 (0)	0.7 (4)
*p* value	0.003	0.001	0.325	0.102	<0.0001	0.132	0.288	0.864

^1^ BRD—bovine respiroatory disease; ^2^ relapse: percentage and number (in parentesis) of animals that showed again BRD after the first episode one (I°), two (II°) or three (III°) times; ^3^ Infirmary = percentage and number (in parentesis) of cattle moved to the infirmary pen; ^4^ Control = traditional nutritional management; Treatment = traditional nutritional management integrated, during the first 30 days after arrival, with a nutraceutical mixture composed by active yeast (LY, 5 g/head/day), organic selenium (organic Se, 3 mg/head/day) and mannan oligosaccharides (MOS, 10 g/head/day).

**Table 2:** Blood parameters indicators of immune functionality in the two experimental groups.

Day	Control ^1^	Treatment ^1^	SEM ^2^	*p* Value
BHV-1 ^3^ serum neutralization, log(dilution)				
d_0_	0.00	0.00	0.745	0.878
d_30_	0.59	0.71	0.045	0.031
Serum bactericidal activity, %				
d_0_	71.23	70.76	0.64	0.608
d_30_	80.86	84.30	0.713	0.0012
γ-interferon, pg/mL				
d_0_	16.73	17.46	0.88	0.559
d_30_	14.20	13.43	0.52	0.302
haptoglobin, mg/mL				
d_0_	0.45	0.39	0.043	0.323
d_30_	0.23	0.19	0.029	0.361
LBP ^4^, ng/mL				
d_0_	7295.17	7010.11	474.03	0.672
d_30_	6342.15	6404.14	489.36	0.928
IL6 ^5^, ng/mL				
d_0_	0.082	0.076	0.016	0.775
d_30_	0.079	0.091	0.017	0.635

^1^ Control = traditional nutritional management; Treatment = traditional nutritional management integrated, during the first 30 days after arrival, with a nutraceutical mixture composed by of active yeast (LY, 5 g/head/day), organic selenium (organic Se, 3 mg/head/day), and mannan oligosaccharides (MOS, 10 g/head/day); ^2^ SEM—standard error of the means; ^3^ BHV-1—bovine herpes virus 1; ^4^ LPB—lipopolysaccharide binding protein; ^5^ IL6—interleukin 6.

**Table 3:** Growth and slaughtering performance in the two experimental groups.

Parameter	Control ^1^	Treatment ^1^	SEM ^2^	*p* Value
Live weight, kg (±ds)				
d_0_	325.47 (±21.44)	323.76 (±23.74)	0.936	0.186
d_30_	349.55 (±20.95)	350.64 (±26.83)	0.941	0.429
d_186_	514.81 (±24.81)	519.01 (±32.40)	1.057	0.006
ADG ^3^, kg/head/day				
ADG_0–186_	1.018 (±0.15)	1.049 (±21.44)	0.023	0.100
ADG_0–30_	0.803 (±0.14)	0.896 (±0.18)	0.007	<0.0001
ADG_30–186_	1.059 (±0.15)	1.079 (±0.12)	0.006	0.034

^1^ Control = traditional nutritional management; Treatment: traditional nutritional management integrated, during the first 30 days after arrival, with a nutraceutical mixture composed by of active yeast (LY, 5 g/head/day), organic selenium (organic Se, 3 mg/head/day), and mannan oligosaccharides (MOS, 10 g/head/day); ^2^ SEM—standard error of the means; ^3^ ADG—average daily gain.

**Table 4:** Carcass characteristics and slaughtering performance in the two experimental group.

Parameter	Control ^1^	Treatment ^1^	SEM ^2^	*p* Value
Cold carcass weight, kg	300.90 (±15.73)	303.68 (±20.95)	0.074	0.600
Dressing percentage, %	58.40 (±0.02)	58.50 (±0.03)	0.0007	0.588
Carcass SEUROP				
% of carcass conformation R- good	9.11	6.06	-	0.172
% of carcass conformation U-very good	86.18	89.36	-	0.172
% of carcass conformation E-excellent	4.76	4.59	-	0.172
% of carcass fatness 2-slight	51.97	48.03	-	0.960
% of carcass fatness 3-medium important	52.11	47.89	-	0.960

^1^ Control = traditional nutritional management; Treatment = traditional nutritional management integrated, during the first 30 days after arrival, with a nutraceutical mixture composed by of active yeast (LY, 5 g/head/day), organic selenium (organic Se, 3 mg/head/day) and mannan oligosaccharides (MOS, 10 g/head/day) ^2^ SEM—standard error of the means.

**Table 5:** Chemical composition of the feces in the two groups.

Parameter	Group		SEM ^2^	*p* Value
	Control ^1^	Treatment ^1^		
Dry matter ^3^, %	20.18	21.07	0.331	0.0570
CP ^4^, % d.m.^2^	13.11	12.96	0.311	0.6857
EE ^5^, % d.m.	2.88	2.97	0.170	0.5499
ADF ^6^, % d.m.	33.11	30.66	0.456	0.0003
NDF ^7^, % d.m.	56.88	53.45	0.540	<0.0001
Starch, % d.m.	8.24	5.11	0.151	<0.0001

^1^ Control = traditional nutritional management; Treatment = traditional nutritional management integrated, during the first 30 days after arrival, with a nutraceutical mixture composed by of active yeast (LY, 5 g/head/day), organic selenium (organic Se, 3 mg/head/day), and mannan oligosaccharides (MOS, 10 g/head/day); ^2^ SEM—standard error of the means; ^3^ d.m.—dry matter; ^4^ CP—crude protein; ^5^ EE—ether extract; ^6^ ADF—acid detergent fiber; ^7^ NDF—neutral detergent fiber.

**Table 6:** Predicted diet composition and nutritional value, calculated by the rationing software (Plurimix).

Feed, kg	Adaptation, d_0_–d_30_	Fattening, d_30_–d_186_
Raw materials, as fed (kg ^1^)		
Wheat bran	0.60	0.60
Hay	1.10	----
Straw	0.80	0.40
Corn silage	8.00	9.50
Corn meal	1.80	2.20
Sunflower meal 28% CP ^2^	0.20	1.20
Soy bean meal 48% CP	0.20	0.50
Mineral and vitamins mix	0.18	0.23
Nutritional values		
As fed, kg	12.78	14.63
d.m. ^3^, kg	6.30	7.80
UFV/kg d.m.	0.89	0.92
CP, % d.m.	12.69	16.44
Lipids, % d.m.	3.57	3.49
Crude fiber, % d.m.	17.71	17.45
NDF ^4^, % d.m.	39.64	34.55
Starch, % d.m.	31.46	33.43
Calcium, % d.m.	0.72	0.85
Phosphorus, % d.m.	0.41	0.57

^1^ kg= kilos; ^2^ CP= crude protein; ^3^ d.m.= dry matter; ^4^ NDF= neutral detergent fiber.

## Data Availability

The data presented in this study are available on request from the corresponding author.
